# United States Veterinary Medical Association

**Published:** 1891-08

**Authors:** W. Horace Hobkins

**Affiliations:** Secretary; 12 So. 37th St, Phila., Pa.


### EDITORIAL DEPARTMENT.

### UNITED STATES VETERINARY MEDICAL ASSOCIATION.

Annual Meeting at Washington, D. C., September 15th 16th, 17th.

All delegates to the Annual Convention of the United States Veterinary Medical Association will be favored with the 1⅓ rates of fare at all points in the Trunk Line Association, Central Traffic Association, Southern Passenger Association, and by the Chicago and Alton R. R. of the Western Passenger Association. They must secure certificates at the point of starting and these will be signed by the Association's Secretary at the meeting which will entitle them to the reduction on the return fare. Further notice will be given as other concessions are granted by other railroads.

The headquarters of the Association will be at Willard's Hotel, where a rate of $3 per day will be granted to all in attendance. The sessions will be held in Willard's Hall, in the hotel building. Delegates will register their names at once with members of the Reception Committee, who will be in attendance. The Veterinarians of Washington and Baltimore will tender a lunch to the members on the opening day of the Convention and will render all aid and information for the benefit of those in attendance.

In addition to the papers, etc. already announced, the Committee of Arrangements have the pleasure of announcing a paper by Dr. W. L. Williams, of Bloomington, Illinois, on "Rachitis". Several other papers are promised for this meeting, and will be announced at a later date.

A trip to Mount Vernon is under consideration by the Committee.

This meeting of the United States Veterinary Medical Association will attract unusual attention from the World of human Medicine and from others, not only from the important subjects which will be discussed, that of food inspection being of as much interest to the human physician as to the veterinarian, but also from the fact that the *Congress of American Physicians and Surgeons*, and the *Association of American Anatomists* will also meet in Washington the following week, September 23rd, 24th, and 25th.

The sessions of the meeting will be closed on the evening of the 17th, by a banquet at Willard's Hotel.

W. Horace Hobkins, *Secretary.*

12 So. 37th St., Phila., Pa.

